# Lessons In Customizing Virtual Communities To Meet The Needs Of Older Adults

**DOI:** 10.1093/geroni/igab046.365

**Published:** 2021-12-17

**Authors:** Lawrence Kosick

**Affiliations:** GetSetUp, Midvale , Utah, United States

## Abstract

GetSetUp started as a technology learning platform for older adults. During Covid-19 we realized that many adults not only wanted to learn technology but were eager for lifelong learning opportunities. Our specially designed technology platform offered a safe, welcoming space that fosters growth, learning, and community for older adults over 60,000 Michiganders over 60 joined in just 4 months. Case studies show how older adults can benefit from technology to feel empowered, reduce social isolation, and improve wellbeing through a job or volunteer opportunities and new friendships. Reports highlight this ongoing need for those with limited community, mobility issues, or those looking to diversify their communities. A trusted virtual community helps connect the physical world digitally to assure older adults understand processes like vaccine enrollment procedures. Virtual communities will not replace physical communities but offer research opportunities, reach more remote communities, and allow for collaboration with state and aging networks.

